# Selective Auditory Attention Associated With Language Skills but Not With Executive Functions in Swedish Preschoolers

**DOI:** 10.3389/fpsyg.2021.664501

**Published:** 2021-05-17

**Authors:** Signe Tonér, Petter Kallioinen, Francisco Lacerda

**Affiliations:** Faculty of Humanities, Department of Linguistics, Stockholm University, Stockholm, Sweden

**Keywords:** language, executive functions, selective attention, early childhood, socioeconomic status, bilingualism, event-related potentials

## Abstract

Associations between language and executive functions (EFs) are well-established but previous work has often focused more on EFs than on language. To further clarify the language–EF relationship, we assessed several aspects of language and EFs in 431 Swedish children aged 4–6, including selective auditory attention which was measured in an event-related potential paradigm. We also investigated potential associations to age, socioeconomic status (SES), bi-/multilingualism, sex and aspects of preschool attendance and quality. Language and EFs correlated weakly to moderately, indicating that relying on measures of vocabulary alone may overestimate the strength of the language–EF relationship. Contrary to predictions, we found no correlations between selective attention and EFs. There were however correlations between morphosyntactic accuracy and selective auditory attention which is in line with previous work and suggests a specific link between morphosyntax and the ability to suppress irrelevant stimuli. In Sweden, socioeconomic differences are rather small and preschool is universally available, but nevertheless, aspects of parental SES predicted children’s performance on all measures. Bi-/multilingual children performed lower on language also when controlling for SES, highlighting the need for interventions to reduce inequalities in educational outcomes already in preschool. A female advantage was found for both language and EFs, whereas preschool attendance and quality were not significantly related to outcome measures. Future work should include longitudinal studies of language and EF development, include children from diverse SES backgrounds and contribute toward a theoretical framework that further clarifies the language–EF relationship.

## Introduction

The development of language skills and executive functions (EFs), including selective attention, seem to be overlapping processes, but the direction and nature of the relationship is still somewhat unclear. Aspects of language skills have been shown to strongly predict later outcomes on an array of domains: literacy, school readiness and psychosocial outcomes (e.g., Justice et al., 2009; Law et al., 2009; Feeney et al., 2012; Duff et al., 2015). Likewise, EFs have predictive value for aspects such as academic achievement, physical health and socioeconomic status (SES; e.g., Moffitt et al., 2011; Stephens et al., 2018). Both language and EF are amenable to improvement (see e.g., Diamond and Lee, 2011; Diamond and Ling, 2016; Grøver et al., 2020; Tarvainen et al., 2020), and improved knowledge about the language-EFs association has possible applications in preschool practices and curricula. However, for typically developing Swedish preschoolers, little is known about the possible relationships between language and EFs and potential differences in these skills due to factors related to the individual and to the environment. Earlier studies from other contexts that have investigated language and EFs have often put EFs in the foreground, conducting an array of EFs tests and experiments but focusing the investigation of language to measures of vocabulary (e.g., Gathercole et al., 1999; Fuhs and Day, 2011; Petersen et al., 2013; Weiland et al., 2014; Miller and Marcovitch, 2015). In our view, empirical investigations of the language-EFs relationship would benefit from a more language-focused approach, and theoretical accounts of the language-EFs association need to more carefully define what is meant by “language,” which in turn would aid in formulating more detailed hypotheses and predictions.

### Language Development

Over the preschool period, children develop their language at an impressive pace, including expanding vocabulary and mastery of morphological and syntactical structures, both receptively and productively (e.g., Tomasello, 2000; Song et al., 2015). The use of language in discourse undergoes rapid development in particular from 3 to 5 years of age, and the ability to tell a story–to construct a narrative–is one aspect of language use that requires and reflects increased linguistic skills as well as cognitive and social skills (e.g., Berman et al., 1994).

Swedish language is an East-Scandinavian language of the North-Germanic branch, and is characterized as a verb-second language, with relatively limited morphology: verbs are not conjugated for person or number and nouns are inflected for number and definiteness only. There are two grammatical genders. For individuals learning Swedish as a second language, word order and noun phrase gender agreement present main challenges (see also Reuterskiöld et al., 2021).

### Executive Functions

There are differing views about the nature of EFs and the debate is ongoing with regard to how to best operationalize these aspects of cognitive control. However, EFs are often described as consisting of three core, interrelated skills: working memory, cognitive flexibility/shifting and inhibition (Miyake and Friedman, 2012; Diamond, 2013), upon which more complex and later-developing skills, such as problem-solving, reasoning, and planning, are developed (Diamond and Lee, 2011). It has been suggested, that EFs are best conceptualized as a unitary construct before school age since EF tasks thought to measure different EF components load onto a common factor in young children (e.g., Wiebe et al., 2008; Fuhs and Day, 2011) but there is no complete agreement, see for instance Howard et al. (2015), for a differing viewpoint. It has furthermore been suggested that a two-factor model with inhibition and working memory as separate dimensions, best describes EFs from age 5 (e.g., Miller et al., 2012), and the authors conclude that the latent structure of EF may depend on the choice of particular tasks and performance indicators.

### Selective Attention

Selective attention can be regarded as either a part of EFs, or as a prerequisite for EFs (see e.g., Diamond, 2013; Dajani and Uddin, 2015). In the former case, selective attention could be reframed as an aspect of inhibition in the form of interference control. Selective attention, or selective information processing, refers to the ability to prioritize relevant stimuli over irrelevant distractors, in other words, to the rather advanced ability to suppress interfering input from complex stimuli (see also Gandolfi et al., 2014). Attention in infancy has been demonstrated to predict EFs in toddlerhood: Frick et al. (2018) found that sustained attention predicted early EF in Swedish infants and toddlers, and authors concluded that early attention is a foundation for EF development. Veer et al. (2017) showed that visual selective attention predicted working memory and inhibition in 2–3-year-olds. Furthermore, selective attention has been proposed to link specifically to the working memory system (Vandierendonck, 2014).

Selective auditory attention is also involved in language processing, specifically so in speech segmentation (Toro et al., 2005) but also in a broader sense: Selective attention helps us communicate in everyday situations in which we need to pay attention to one speaker in the presence of distractors, and to dynamically redirect attention to different speakers or other sources of auditory information (e.g., Shinn-Cunningham and Best, 2015). Neural correlates of auditory sustained selective attention has been investigated with behavioral methods but also in experimental designs using event-related potentials (ERPs), starting with classic dichotic listening experiments on adults (e.g., Hillyard et al., 1973) as well as ERP paradigms adapted for young children (e.g., Coch et al., 2005; Sanders et al., 2006; Stevens et al., 2009).

### The Language–EF Relationship

Some studies have indicated that aspects of EFs seem to lay the foundation for aspects of language development, leading to the assumption that good EFs facilitate language learning (e.g., Weiland et al., 2014; Woodard et al., 2016; ten Braak et al., 2018). On the other hand, language has been claimed to play a crucial role in the development of EFs (e.g., Kuhn et al., 2014; Miller and Marcovitch, 2015; Botting et al., 2017). It has also been suggested that the relation between language and EFs is dynamic and may depend upon the specific skills investigated and when during development these skills are assessed (e.g., Friend and Bates, 2014; Bohlmann et al., 2015; Slot and von Suchodoletz, 2017). The lack of consensus regarding the language–EF relationship is in turn related to the lack of a universally accepted theory of EFs and, possibly, to vague and/or limited definitions and operationalizations of language. The investigation of relationships between aspects of language and specific EF components is also obstructed by the lack of clarity regarding the latent structure of EF in early childhood, as mentioned above.

Examples of existing EF theories, which to some extent include language include Barkley’s (1997) suggestion that internal speech should be considered as an EF, and Zelazo’s (2015) suggestion that EFs are verbally mediated and that EF development involves the improvement of formulating increasingly complex hierarchical rules. For Barkley’s theory, it is unclear exactly what such an idea would entail in terms of predicting more specific aspects of the language-EF relationship, see also Jones (2009) for a critical appraisal of internal speech as a concept. If Zelazo’s idea holds, one would predict stronger associations between EFs and syntactic skills compared to other aspects of language, since syntax is concerned with embedded, rule-governed structures, and one would expect language measures to predict EF better than vice versa. Results pointing to the crucial role of language for EF development include a study by Botting et al. (2017) who examined language and EFs in deaf children and found that language mediated (non-verbal) EFs but not vice versa, suggesting that language is key to EF.

However, an opposite direction of the relationship is also suggested, in other words that aspects of EF are involved in language processing. To the extent that inhibitory processes can be reliably isolated in early childhood, aspects of inhibition in particular have been shown to associate with language. Gandolfi and Viterbori (2020), hypothesized that inhibition would be important in language acquisition by enabling children to deal with interfering information during sentence processing, and results suggested that interference suppression could be involved in both lexical production and expressive grammar in preschool-aged children. Kaushanskaya et al. (2017) showed that non-verbal inhibition predicted school-aged children’s syntactic abilities. For Swedish preschool-aged children, Tonér and Nilsson Gerholm (2021), found concurrent associations between measures of inhibition and morphosyntactic accuracy. Woodard et al. (2016) showed that inhibition plays a role in young children’s interpretation of ambiguous sentences. Furthermore, findings by Friend and Bates (2014) indicate that the ability to maintain focus and inhibit prepotent responses at 4 years of age supports subsequent narrative ability, and Blain-Brière et al. (2014) showed that EFs contributed more than IQ to typically developed preschoolers’ pragmatic skills during conversation.

With regard attention in a broad sense, D’Souza et al. (2017) proposed that infants’ ability to allocate attention may be crucial for them to attend to important linguistic input, which in turn would affect language development – in other words, attentional capacities is one of several possible constraints on language development. If on the other hand some aspect of language aids selective auditory attention specifically, one might predict that language-focused intervention would improve attention, which has actually been shown to be the case: auditory selective attention was improved after vocabulary training (Stevens et al., 2008) and after intervention targeting early literacy (Stevens et al., 2011). However, it is still unclear what constitutes the mechanism behind the gains in attention, and it is theoretically possible that the language and literacy interventions also included aspects targeting attention specifically.

There is a risk that tests that purport to assess EFs, actually also place high demands on language. Even “non-verbal” EF tests often require at least some level of language comprehension, something that is seldom mentioned or problematized in the literature on EFs (see Deák, 2014; Kaushanskaya et al., 2017 for a discussion). Conversely, language tests often require some EFs. There is in other words a potentially large task impurity problem which needs consideration when selecting tasks and interpreting the results. It could also be argued that associations between pragmatic abilities and EFs could be regarded as trivial: Emerging pragmatic skills, including children’s narrative ability, involve both linguistic, social, and cognitive abilities (e.g., Berman et al., 1994; Fernández, 2011), However, finding spurious relationships between narrative ability and EFs would probably be more likely when examining narratives with respect to overall coherence than when extracting information regarding content, syntax and vocabulary from the narratives.

### Demographic Factors

Development is constrained both by our biological heritage and by factors in human environments. It is well established that SES is connected to children’s acquisition of language and EFs skills, including auditory selective attention (e.g., Hoff, 2003; Stevens et al., 2009; Sarsour et al., 2010; Ursache and Noble, 2016). In Sweden, the socioeconomic differences are smaller than in most other OECD countries despite a rapid surge of income inequality since the early 1990s. Poverty rates are among the lowest, 28% of the population have higher education, women have a high employment rate compared to other OECD countries and unemployment is receding, although it remains high for foreign-born (OECD, 2017; SCB, 2017, 2018). The association between SES and language/EFs/attention could thus be expected to be weaker in Sweden than in contexts with larger socioeconomic differences and more unequal access to high quality child care.

In Swedish preschools, 25% of children are either born in another country or have two parents that are foreign-born and are thus likely to be dual language learners (Puskás and Björk-Willén, 2017). Increased variability in majority language skills may be a result of variations in exposure, which in turn could be related to age at preschool start (children to foreign-born parents start preschool later than children to Swedish-born parents), and the possibility to use the majority language in an array of communicative contexts. It has been shown in a large sample of German preschoolers that high preschool quality seems to be extra important for dual language learners with low exposure to the majority language (e.g., Kohl et al., 2019). Calvo and Bialystok (2014) showed in a sample of Canadian children that bilingual children performed lower than monolingual children on language tasks in the majority language also when taking SES into account. However, a small Swedish study indicated that there were no significant differences in language skills when comparing monolingual and bi-/multilingual children (Tonér and Nilsson Gerholm, 2021). Bi- or multilingual children have often been reported in the literature to perform better with regard to EFs than monolinguals (e.g., Adesope et al., 2010; Calvo and Bialystok, 2014; Barac et al., 2016). However, Duñabeitia et al. (2014) conducted a large-scale study with school-aged children and adolescents and found no support for a bilingual advantage. A small Swedish study did not find any differences in EFs between monolingual and multilingual children (Tonér and Nilsson Gerholm, 2021) and a meta-analysis has indicated that cognitive advantages related to bi-/multilingualism may be a result of publication bias (de Bruin et al., 2014). With regard to possible differences between girls and boys in language skills, previous results are diverging. Eriksson et al. (2012) found a female language advantage in a large sample of children aged 8–30 months across 10 language communities, including Sweden, indicating that sex-related language differences can be detected from an early age. For EFs, previous work regarding associations to sex is inconsistent. On one hand, girls have outperformed boys with regard to EFs in a number of studies (e.g., Fuhs and Day, 2011; Mulder et al., 2014). On the other hand, no EF or language differences were found in a sample of German children aged 3–4 (Slot and von Suchodoletz, 2017), a cross-cultural study including French, German and Icelandic children found no sex-related EFs differences (Gestsdottir et al., 2014) and a recent review concluded that there is little support for significant sex-related differences in EFs (Grissom and Reyes, 2019).

A vast majority of Swedish children attend preschool more or less full-time from an early age, and in the age range 4–6, over 95% of children attend preschool (The Swedish National Agency for Education, 2019). Fees are heavily subsidized, and there is a national curriculum for the preschool, intending to guarantee that quality is equally high in all preschools. However, audits and reports during recent years (e.g., The Swedish Schools Inspectorate, 2018) have shown that this is not the case, prompting the Swedish parliament to call for a thorough investigation of the conditions for an equivalent and sustainable preschool.

## Current Study

### Aims and Research Questions

There is a need for a better understanding of the relationships between language skills, EFs and auditory selective attention and of the potential links between these measures and factors relating to the individual and the environment. In the current study, potential links between diverse measures of language, EFs and auditory selective attention are investigated, as well as possible links between these measures and age, SES and multilingualism. Additionally, we explore potential differences between girls and boys with regard to language EFs and selective attention as well as potential associations to preschool quality.

RQ1. What is the relationship between different language skills, EFs and auditory selective attention in a sample of Swedish preschoolers?
RQ2. Do age, SES, sex, bi/-multilingualism, and aspects of preschool attendance and quality make significant contributions in explaining language/EFs/selective attention variance?

The first research question is addressed by applying descriptive methods. We expect that language skills and EFs will be significantly correlated in Swedish preschoolers, similar to previous findings in other populations and that correlations will be at least moderate in magnitude. We predict an association between behaviorally assessed EF and auditory selective attention measured with ERPs, based on assumptions that selective attention is either a prerequisite for or an intrinsic part of EFs (e.g., The second research question is addressed by fitting multiple regression models. We hypothesize that child age and aspects of family SES will explain unique variance in language/EFs/selective attention. With regard to associations to sex. bi-/multilingualism and preschool quality, we refrain from formulating any hypotheses, since previous research is diverging and/or scarce.

## Materials and Methods

### Participants

Ethics approval for this project was granted by the regional ethical review board^1^ and data were treated in accordance with the EU General Data Protection Regulation. Data for the current study were collected within the framework of an intervention study aimed at all children in 18 preschools from a municipality in the Stockholm region (Gerholm et al., 2018, 2019). The proportion of trained preschool teachers was 27%, whereas the national average was 39% at the point of data collection (The Swedish National Agency for Education, 2016). All children whose caregivers gave written consent were considered eligible for participation. The children were informed about the study, including their right to withdraw at any time. Participants did not receive any compensation for participating in the study. The sample consisted of 431 children aged 44–74 months (*M* = 62, SD = 7; 52% girls),. Children came mainly from higher-SES backgrounds; 65% had at least one parent with university level education. They spent on average 38 h per week at preschool and had started preschool at on average 18 months of age; 90% of participants were enrolled in preschool at 2 years of age or younger. Bi-/multilingual children composed 33% of the final sample and 43 different languages were represented. English (*n* = 24), Arabic (*n* = 12), Spanish (*n* = 12), Polish (*n* = 10), and Kurdish (*n* = 8) were the most frequent languages spoken in the home apart from Swedish, and in 40 cases, parents reported that Swedish was not the child’s strongest language. According to parental reports, 29 children (12 girls), corresponding to 7% of the sample, had a language disorder, largely in line with the prevalence of language disorders in the population (e.g., Tomblin et al., 1997). Children with language disorders did not differ from children with reported typical language development with regard to age or SES.

### Materials

#### Language

In terms of language assessment, narratives provide rich information concerning form, content, and use of language with little risk of ceiling effects even when collecting data from children of various ages. The *Bus Story Test* (*BST*; Renfrew, 1995; Svensson and Tuominen-Eriksson, 2002) was used to elicit narratives. The child first listens to a story told by the examiner, then retells the story, aided by picture prompts. The children also completed the *Peabody Picture Vocabulary Test (PPVT-IV)*, which assesses receptive vocabulary (Dunn and Dunn, 2007). The examiner says a word and the child’s task is to indicate which out of four alternatives presented on a picture plate best resembles the meaning of that word. Since there is neither an authorized Swedish translation nor Swedish norms available for the PPVT, only raw scores were used. Parents completed a preliminary Swedish version of the *McArthur-Bates communicative development inventories* (SCDI-III) for children aged 30–48 months (Eriksson, 2017), rendering information about parents’ perceptions of their child’s expressive vocabulary and morphology. SCDI-III norms do not cover the age span in the current sample and results were treated with caution.

#### Executive Functions

The *Dimensional Change Card Sort* (*DCCS*) primarily assesses the ability to flexibly switch between rules (Doebel and Zelazo, 2015). The child sorts pictures according to the shape of the objects (pre-switch phase, 5 items) and then switch to a new rule and instead sort by color (post-switch phase, 5 items). In the final stage of the task, the child needs to alternate between these two sorting strategies (mixed trials, 30 items). Scoring is done automatically via the application and is based on a combination of accuracy and reaction time. For any given individual, accuracy is first considered, and if accuracy levels are ≤ 80%, the final score is equal to the accuracy score. Reaction times are log transformed to create a more normal distribution (for full details of scoring, see Slotkin et al., 2012). The *Fish Flanker task* mainly taps into the ability to disregard irrelevant visual stimuli and the test requires children to indicate the direction of a central stimulus flanked by congruent or incongruent flankers (Rueda et al., 2012). For children aged 3–7, 20 trials with fish stimuli are conducted. If performance is ≥ 90%, 20 additional trials with arrows are presented. The two tests mentioned above were delivered via a tablet application, but instructions were given by the examiner, since no Swedish-speaking version of the tablet application is available. Scoring is completed automatically in the application and is identical for DCCS and the flanker task. However, for children who do not proceed to the arrow trials in the flanker task, reaction time is not considered (Slotkin et al., 2012; Weintraub et al., 2013). *Forward and Backward digit span* (FDS and BDS), assesses short term memory and working memory in the auditory-verbal modality (Gathercole et al., 1999). The *Head-Shoulders-Knees-and-Toes task (HTKS)*, places demands both on inhibitory control and working memory (Cameron Ponitz et al., 2008). The child is first instructed to touch his/her toes when the examiner says “Touch your head!” and vice versa. In the second phase, the child is instructed to touch his/her knees when the examiner says “Touch your shoulders!” and vice versa, and in the third phase, all four instructions are included.

#### Selective Auditory Attention

A Swedish adaptation of a dichotic listening ERP paradigm (e.g., Coch et al., 2005; Stevens et al., 2009; Neville et al., 2013) was used, henceforth referred to as AudAt. The child was instructed to pay attention to one of two simultaneously played stories and the attention effect was measured as the difference between the average response to attended and unattended probe sounds. The task could thus be described as tapping into selective, sustained auditory attention.

#### Background Information

Parents provided information via a questionnaire about the child’s age, family background, medical conditions, heredity for language or reading difficulties, languages spoken at home as well as income and educational level. There were three income categories, where low and high income corresponded to approximately the 10th and 90th percentiles in the Swedish population. There were four educational level categories: elementary school, upper secondary school, vocational education and college/university education. See also Table 1. Parents also gave information regarding their child’s age at preschool enrollment and current amount of preschool time/week. Questionnaires including background information, medical history, and SCDI-III were administered to parents in paper versions via the preschools and returned in prepaid envelopes. For every preschool unit/classroom, quality was rated with Early Childhood Environmental Rating Scale (ECERS-3, Harms et al., 2014) by researchers with extensive experience with the instrument. The full ECERS scale was used, encompassing information regarding preschool space and furnishings, care, language and literacy, play and learning, interaction, and organization. *Z*-scores were used in further analysis.

**TABLE 1:** Raw scores for the language, EF, and selective attention measures.

	Mean	SD	Range	First quartile	Third quartile
**Language**					
Information* (*n* = 384)	17.74	9.64	0–44	10	24.25
Syntactic complexity* (*n* = 383)	2.40	2.19	0–13	1	4
Unified predicates* (*n* = 384)	16.73	6.88	0–35	12	21
Morphosyntactic accuracy* (*n* = 384)	0.64	0.24	0–1	0.50	0.81
Receptive vocabulary** (*n* = 395)	79.19	30.73	0–129	62	100
SCDI vocabulary*** (*n* = 404)	82.61	14.10	0–100	76.30	93.00
SCDI morphology*** (*n* = 398)	8.29	2.24	0–11	7.00	10.00
**EF**					
DCCS (*n* = 377)	4.20	1.40	0.13–7.83	3.38	5.0
Flanker (*n* = 371)	4.35	1.67	0.13–8.78	3.13	5.56
FDS (*n* = 380)	4.56	1.73	0–10	4	6
BDS (*n* = 367)	1.17	1.41	0–5	0	2
HTKS (*n* = 386)	15.5	7.93	0–24	10	22
**Selective attention**					
Early attention effect (*n* = 106)	0.69	2.28	−5.57 to 6.98	−0.78	2.37
Late attention effect (*n* = 108)	−0.28	2.08	−5.03 to 5.75	−1.61	1.09

Number of respondents for each measure within parentheses. *The measure was extracted from transcripts of the Bus Story narratives. **Receptive vocabulary was based on results from the PPVT. ***SCDI measures were based on parental questionnaires.

### Procedure

#### Behavioral Measures

Language and EF testing was conducted in two sessions by trained research assistants on-site at the preschools during a 2-week period. Each session lasted 20–40 min. All behavioral testing was audio- and video recorded to enable multimodal annotation and to double-check examiners’ adherence to protocol. The tasks were presented in a predetermined order to provide sufficient variation for the participants and to control session duration, based on a pilot study (Tonér and Nilsson Gerholm, 2021). The order of presentation for the first session was DCCS, Test of Emotion Comprehension (not further reported here), BST, a math task (not further reported here) and HTKS. The order for the second session was the Flanker task, PPVT, and finally the digit span tasks.

#### Event-Related Potential Recording

**FIGURE 1:**
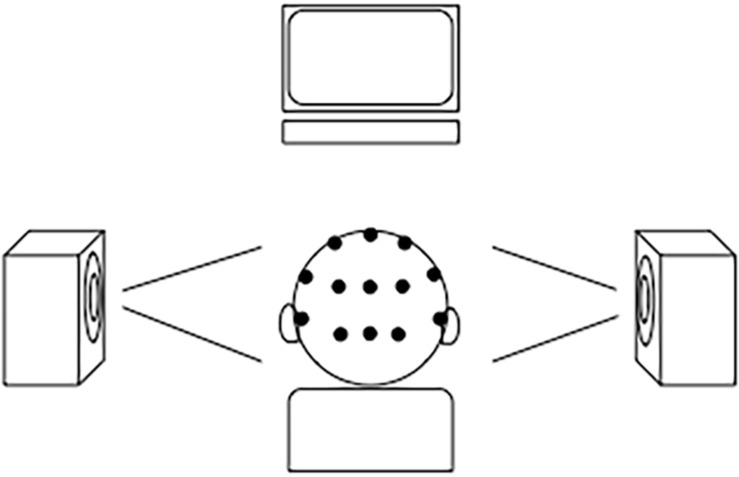
Experimental setup of Swedish AudAt.

**FIGURE 2:**
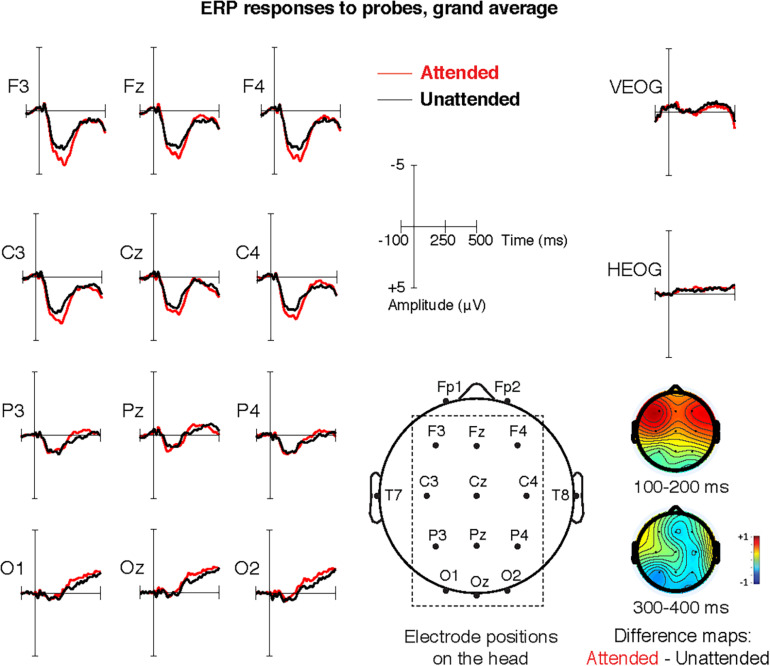
Grand average ERP responses to probes in the attended and unattended channel and electrode placement in Swedish AudAt. Topographic maps show differences in amplitude between attended and unattended in the early and the late time window respectively.

AudAt was conducted on-site on a randomized subsample representing all preschool units and consisting of 138 children (75 girls). Selection was based on a randomized priority list so that if a child declined to participate, the next child on the list would be asked instead. Recordings took place during the same 2-week period as the behavioral testing and were conducted by the first and second author. EEG was recorded using a BioSemi activeTwo amplifier with 16 head channels and a Common Mode Sense/Driven Right Leg (CMS/DRL) loop in a cap, two external mastoid channels and four external eye channels^2^. The child was seated on a small chair with speakers 0.7 m from each ear to the left and to the right. The child was informed about the experiment (information had also been given previously) and cap and electrodes were applied (for experimental setup, see Figure 1; for electrode placement, see Figure 2).

Probe sounds in the form of the syllable “Ba” and a “Bz”-like noise were embedded in two simultaneously played stories, that differed by content, by gender of the reader’s voice and by presentation to the left or right. The “Bz” noise was constructed by splicing 20 ms segments of “Ba” and then scrambling all segments except the first and the last. The procedure resulted in a broad-spectrum “Bz” that preserved many of the acoustic properties of the linguistic “Ba” probe but at the same time sounding non-linguistic (see also e.g., Stevens et al., 2011). Both types of probes had a duration of 200 ms and were presented randomly in both channels at inter-stimulus intervals of 200, 550, or 1,000 ms. The child’s task was to attend to one story while ignoring/suppressing the other, and images from the attended story were displayed on a laptop 1.0 m in front of the child to further aid selective attention. Each recording session involved two pairs of stories, with comprehension questions after each story pair, and lasted 20–40 min, including application and de-application, see also Gerholm et al. (2019).

### Data Processing

Care was taken to ensure the anonymity of participants. All test protocols, test data achieved by the tablet application, teacher and parental questionnaires and ECERS data were coded by a researcher not directly involved in data collection or statistical analysis. The code key was not known to any of the authors (see also Gerholm et al., 2018).

#### Behavioral Measures

##### Language

The bus stories were orthographically transcribed and annotated in ELAN (Max Planck Institute for Psycholinguistics, The Language Archive, Nijmegen, The Netherlands; Wittenburg et al., 2006^3^) and a number of language measures were extracted. Two of those were based on the BST manual (Renfrew, 1995; Svensson and Tuominen-Eriksson, 2002), namely information score and, as a proxy for syntactic complexity, the number of subordinate clauses. Information score concerns the information density in the retell and that children include relevant content, correct sequencing of those events and provide appropriate amount of context; a scoring guide for Swedish is provided in the test manual. In addition, we extracted a measure of text length, counted as number of unified predicates (e.g., Berman, 1988), and a measure of morphosyntactic accuracy, an often-used measure in first as well as second language acquisition (e.g., Zwitserlood et al., 2015; Meir, 2018), here operationalized as the proportion of morphosyntactically well-formed utterances (see also Tonér and Nilsson Gerholm, 2021, for results regarding Swedish children). Raw scores from the PPVT were used to represent a crude measure of receptive vocabulary.

##### Executive functions

In addition to examining correlations to language and attention for the separate EF tasks, raw scores from DCCS, the Fish Flanker task, digit span and the HTKS tasks were z-transformed and summed to a composite EF score with a mean of 0 and a standard deviation of 1. The composite EF measure was used in regression models since the suggested EF components are hard to measure in isolation and since it has been argued that the components cannot be clearly separated for the current age span.

#### Event-Related Potential Data

Data processing was done in EEGLAB (Delorme and Makeig, 2004). Sampling rate during recording was 2 KHz, downsampled to 256 Hz offline, re-referenced to average mastoids and filtered with a band pass filter of 0.1 and 40.0 Hz. Bad channels were identified visually, removed and interpolated. The continuous data was epoched with respect to probe sound onsets (100 ms before stimulus onset to 500 ms after stimulus onset). Artifacts were first automatically rejected by using the ERPLAB moving window peak-to-peak artifact detection algorithm (Lopez-Calderon and Luck, 2014), removing epochs with head channel amplitudes larger than +200/−200 μV or eye channel amplitudes larger than +100/−100μV across a 200 ms time window, moving at 50 ms increments. Thereafter, EEG data was visually inspected by the first and second author and residual artifacts were removed manually (see also Stevens et al., 2009). The rejection rate was on average 45%. Complete exclusion of 29 recordings was necessary due to noisy or flat average response and/or less than 100 epochs remaining after artifact rejection. Following the original AudAt studies as well as the analytic procedure in an unpublished pilot study on Swedish AudAt, mean amplitudes relative to baseline were measured between 100 and 200 ms post-stimulus onset. Any difference in amplitudes in this time window, between responses to attended and unattended probes, constitute the early attention effect. Additionally, a separate analysis was conducted of the attention effect in a later time window, at 300–400 ms post-stimulus onset. There were 19 children who failed to answer any of the comprehension questions correctly. Previous studies using the original AudAt paradigm have used a cutoff of at least 50% correctly answered comprehension questions to include children’s ERP data in further analysis (Stevens et al., 2009; Neville et al., 2013; Karns et al., 2015; Hampton Wray et al., 2017). In an early study, Coch et al. (2005) used a cutoff of 8/10 correctly answered comprehension questions but commented that this procedure may have biased their sample. For the current study, we decided not to exclude children based on results on comprehension questions. The expected difference in response to attended versus unattended stimuli is considered pre-linguistic, and electrophysiological signs of selective auditory attention should thus not be dependent upon language comprehension. Furthermore, there was no significant difference in attention, neither in the early nor in the late time window, between children who passed comprehension questions and those who failed to answer any question correctly.

#### Questionnaires

Background information and SCDI questionnaires were already anonymized when arriving by post to the handling researcher. Data thereof were connected to behavioral and ERP data via individual codes (see also Gerholm et al., 2019). Raw scores from SCDI subscales for vocabulary and morphology were used in analysis.

## Results

Data were analyzed with R software (Version 3.7.0; R Core Team, 2019). There are missing values for separate test measures due to children declining to participate and due to technical problems, see also Table 1 for number of respondents for each measure.

### RQ1: Associations Between Language and EFs

An overview of children’s performance on separate language and EF measures is provided in Table 1. Children who gave some verbal output, for instance in form of one-word and/or elliptical utterances in the narrative task were included in analysis, which entails that a score of 0 is possible for several of the language measures. Non-parametric correlations were calculated since some tests/tasks did not fulfill the requirements for parametric testing. See Table 2 for all significant correlations. There were strong correlations between all language measures extracted from the narratives. Correlations between receptive vocabulary (PPVT score) and the other language measures were moderate in strength (ρ ranging from 0.38 to 0.57, *p* < 0.001). Additionally, parents’ ratings of children’s vocabulary skills and morphology with SCDI-III were weakly to moderately correlated with behaviorally assessed language. All EF measures correlated significantly with one another (*p* < 0.001), but the correlations were moderate at best, the strongest correlations were found between the Flanker task and BDS (ρ = 0.47), between DCCS and HTKS (ρ = 0.45) and between HTKS and BDS (ρ = 0.43).

**TABLE 2:** Significant Spearman correlations for language, EF, and selective attention measures.

Measure			1	2	3	4	5	6	7	8	9	10	11	12	13	14
Bus Story Test	1	Information	–													
	2	Syntactic complexity	0.67	–		.										
	3	Unified predicates	0.80	0.73	–											
	4	Morphosyntactic accuracy	0.77	0.67	0.94	–										
PPVT	5	Receptive vocabulary	0.57	0.41	0.40	0.38	–									
SCDI	6	Expressive vocabulary	0.28	0.25	0.20	0.19	0.34	–								
	7	Expressive morphology^*c*^	0.32	0.26	0.23	0.21	0.38	0.45	–							
DCCS	8	EFs; cognitive flexibility	0.33	0.24	0.23	0.22	0.42	0.22	0.26	–						
Flanker Fish Task	9	EFs; inhibition	0.33	0.25	0.18	0.15	0.44	0.17	0.31	0.42	–					
FDS	10	EFs; short-term/working memory	0.31	0.17	0.23	0.25	0.31	0.16	0.19	0.30	0.29	–				
BDS	11	EFs; working memory	0.43	0.22	0.29	0.26	0.56	0.27	0.36	0.36	0.47	0.41	–			
HTKS	12	EFs; inhibition, working memory	0.42	0.30	0.25	0.19	0.49	0.26	0.26	0.45	0.36	0.39	0.43	–		
AudAt	13	Early attention effect			0.24	0.27									–	
	14	Late attention effect													0.43	–

*All correlations were significant at *p* < 0.001 except associations between morphosyntactic accuracy and the flanker task, morphosyntactic accuracy and early attention effect, SCDI vocabulary and FDS, SCDI vocabulary and the Flanker task (*p* < 0.01), and between early attention effect and unified predicates (*p* < 0.05).*

As for associations between language and behaviorally assessed EFs, all measures correlated weakly to moderately, the strongest correlations were found between EF measures and PPVT (see Table 2). The SCDI measures also showed significant but overall weak correlations with EFs. With regard to auditory selective attention, the magnitude of the attention effect in the early time window (100–200 ms) correlated with the number of unified predicates (ρ = 0.24, *p* < 0.05), and with morphosyntactic accuracy (ρ = 0.27, *p* < 0.01). In other words, children who told longer stories and who had a higher ratio of correct utterances had a larger early attention effect. Selective auditory attention in the early time window did not correlate with any other language or EF measure. The late time window attention effect did not correlate with any language or EF measures. See Figure 2 for ERP responses to attended and unattended probes.

### RQ2: Associations to Background Factors

Multiple linear regression models were fitted with the lm function (R Core Team, 2019) to investigate whether background factors significantly predicted language, EF and auditory selective attention measures. Morphosyntactic accuracy represents children’s productive grammar abilities, whereas the PPVT score represents receptive vocabulary and the EF composite score represents EFs. Included predictors were based on the hypotheses of the current study. The role of SES was investigated by including educational level and income separately for each parent^4^. Effects of being a dual language learner were explored by including multilingualism as a predictor but also including information on whether or not Swedish was the child’s stronger language, as judged by parents. Possible effects of preschool-related factors were explored by including age at preschool enrollment, current time/week at preschool and preschool quality assessed with ECERS-3 as predictors. All models controlled for age. A backward elimination procedure was employed, in each step removing the least contributing predictor, and models which could explain as high proportion of variance as possible with as few predictors and as low residual standard error as possible, were preferred.

#### Receptive Vocabulary (PPVT)

**FIGURE 3:**
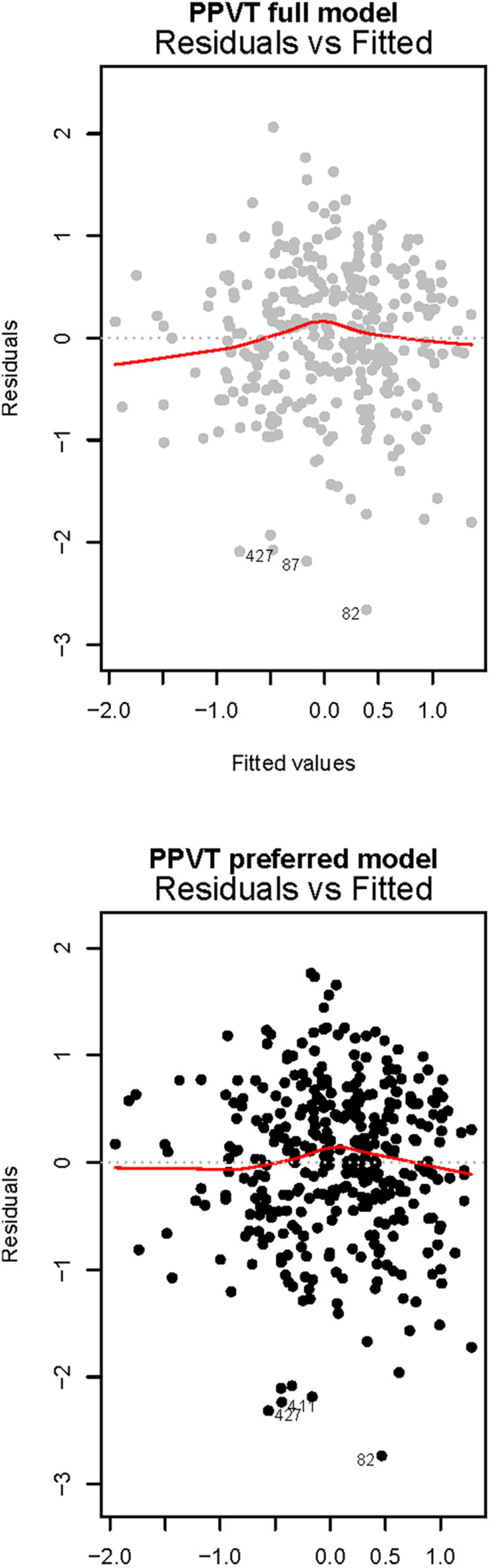
Residuals versus fitted plots of receptive vocabulary regression models. The full model for receptive vocabulary included all predictors and explained 41% of PPVT score variance, whereas the preferred model included only significant predictors, explaining 40% of PPVT variance.

Two models explained very similar levels of PPVT score variance (Table 3). The preferred model included only significant predictors and explained 40% of PPVT variance. Age, having Swedish as a stronger language, and higher parental SES (both education and income) positively predicted PPVT score, whereas being a boy and being multilingual were significant negative predictors of children’s receptive vocabulary. See also Figure 3 for residuals versus fitted plots of receptive vocabulary regression models.

**TABLE 3:** Model comparison for PPVT score.

Predictor	Full PPVT model			Preferred PPVT model			
	Adjusted *R*^2^ = 0.41			Adjusted *R*^2^ = 0.40			
	RSE = 0.72 (300 DF)			RSE = 0.73 (354 DF)			
	*p* < 0.0001			*p* < 0.0001			
	β	SE	*p* <	β	SE	*p* <	95% CI
Intercept	−6.77	0.67	0.0001	−5.89	0.49	0.0001	−6.84 to −4.93
**Age**	0.07	0.006	0.0001	0.07	0.006	0.0001	0.05–0.08
**Boy**	−0.18	0.08	0.05	−0.16	0.08	0.05	−0.31 to −0.005
**Multilingual**	−0.24	0.10	0.05	−0.32	0.09	0.001	−0.50 to −0.14
**Swedish stronger language**	0.58	0.17	0.001	0.70	0.15	0.0001	0.40–1.0
**Education parent 1**	0.09	0.03	0.01				
**Education parent 2**	0.02	0.03		0.07	0.02	0.01	0.02–0.35
**Income parent 1**	0.17	0.08	0.05	0.21	0.07	0.01	0.08–0.35
Income parent 2	0.10	0.09					
Preschool time/week	0.0003	0.007					
Age at preschool start	0.001	0.008					
Preschool quality	−0.05	0.04					

*Standardized estimates, standard errors, and significance levels for predictors are included, as well as 95% confidence intervals for predictors in the preferred model. Adjusted *R*^2^ and residual standard error (RSE) displayed for the full model and the preferred model. Significant predictors in bold script.*

#### Morphosyntactic Accuracy

No model provided a good fit to the morphosyntactic data, see Table 4 for model comparison. A reduced model including age, sex, education in one parent, income in both parents, age at preschool enrollment, time/week at preschool and preschool quality explained 13% of variance and had a slightly lower residual standard error than the full model and any intermediate models. Age and parental education were significant positive predictors of morphosyntactic accuracy whereas male sex was a negative predictor. Further reduction of the model rendered lower levels of explained variance.

**TABLE 4:** Model comparison for morphosyntactic accuracy.

Predictor	Full morphosyntax model			Preferred morphosyntax model			
	Adjusted *R*^2^ = 0.12			Adjusted *R*^2^ = 0.13			
	RSE = 0.89 (293 DF)			RSE = 0.89 (298 DF)			
	*p* < 0.0001			*p* < 0.0001			
	β	SE	*p* <	β	SE	*p* <	95% CI
Intercept	−3.43	0.83	0.0001	−3.54	0.79	0.0001	−5.09 to −1.99
**Age**	0.04	0.008	0.0001	0.04	0.008	0.0001	0.03–0.06
**Boy**	−0.28	0.10	0.01	−0.27	0.11	0.01	−0.48 to −0.07
Multilingual	0.05	0.12					
Swedish stronger language	0.05	0.22					
**Education parent 1**	0.09	0.04	0.05	0.09	0.04	0.05	0.02–0.17
Education parent 2	−0008	0.04					
Income parent 1	0.17	0.10		0.17	0.10		
Income parent 2	−0.05	0.11		−0.06	0.10		
Preschool time/week	−0.008	0.009		−0.008	0.009		
Age at preschool start	−0.009	0.01		−0.01	0.009		
Preschool quality	0.06	0.05		0.05	0.05		

*Standardized estimates, standard errors, and significance levels for predictors are included, as well as 95% confidence intervals for predictors in the preferred model. Adjusted *R*^2^ and residual standard error displayed for the full model and the preferred model. Significant predictors in bold script.*

#### Executive Functions

**FIGURE 4:**
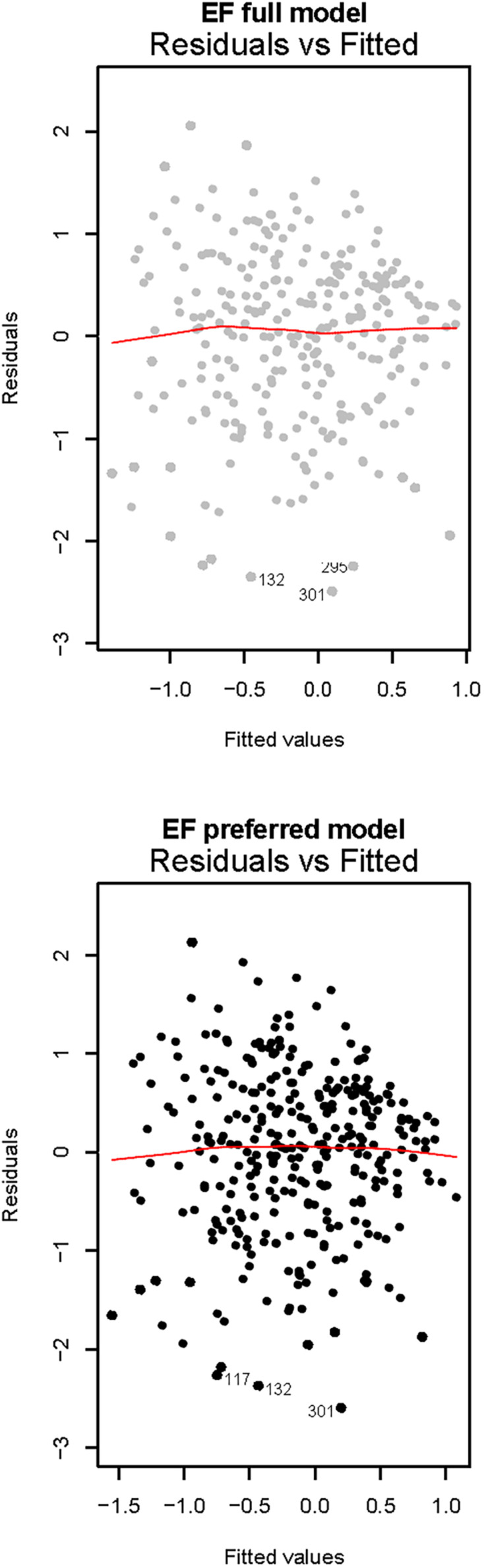
Residuals versus fitted plots of EF regression models. The full model for EF included all predictors and accounted for 26% of EF score variance, whereas the preferred model included age, sex, parental education, and age at preschool enrollment, and explained 29% of EF score variance.

Two models explained similar levels of variance, see Figure 4 and Table 5 for model comparison. The preferred model included age, sex, educational level in parent 1 and age at preschool enrollment and explained 29% of EF score variance. Age and parental education were highly significant positive predictors, male sex was a highly significant negative predictor and age at preschool enrollment was a negative, albeit not significant predictor of EF score (*p* = 0.05). Since EF scores were *z*-transformed, the results can thus be interpreted as follows: when keeping all other variables constant at their mean, male sex corresponded to a decrease in EF score of −0.33 SD.

**TABLE 5:** Model comparison for EF score.

Predictor	Full EF model			Intermediate EF model			Preferred EF model			
	Adjusted *R*^2^ = 0.26			Adjusted *R*^2^ = 0.28			Adjusted *R*^2^ = 0.29			
	RSE = 0.84 (258 DF)			RSE = 0.82 (294 DF)			RSE = 0.83 (315 DF)			
	*p* < 0.0001			*p* < 0.0001			*p* < 0.0001			
	β	SE	*p* <	β	SE	*p* <	β	SE	*p* <	95% CI
Intercept	−6.03	0.84	0.0001	−6.03	0.69	**0.0001**	−5.91	0.62	0.0001	
**Age**	0.07	0.008	0.0001	0.07	0.007	**0.0001**	0.07	0.007	0.0001	0.06–0.08
**Boy**	−0.33	0.10	0.01	−0.34	0.1	**0.001**	−0.33	0.09	0.001	−0.5 to −0.14
Multilingual	0.003	0.13								
Swedish stronger language	−0.13	0.22		−0.13	0.19					
**Education parent 1**	0.10	0.04	0.05	0.10	0.04	0.01	0.13	0.03	0.0001	0.07–0.19
Education parent 2	0.03	0.04		0.03	0.04					
Income parent 1	−0.005	0.1								
Income parent 2	0.07	0.11		0.06	0.09					
Preschool time/week	0.002	0.009	−							
Age at preschool start	−0.007	0.01	−	−0.01	0.008		−0.02	0.008		
Preschool quality	0.01	0.06	−	−0.02	0.05					

*Standardized estimates, standard errors, and significance levels for predictors are included, as well as 95% confidence intervals for predictors in the preferred model. Adjusted *R*^2^ and residual standard error displayed for the full model and the preferred model. Significant predictors in bold script.*

#### Selective Auditory Attention

The full model for the early attention effect (in other words, the mean difference in brain responses for the attended story versus the unattended story in the time window between 100 and 200 ms post stimulus onset) with all background variables as predictors was not significant. Significance was first reached with a model including age, Swedish as the stronger language, education and income levels for both parents, however explaining only 9% of variance in the early attention effect, see Table 6. Having Swedish as a stronger language was a negative predictor of early attention effect. Eliminating the least contributing factor (income in parent 1) did not improve the model (adjusted *R*^2^ = 9%) but education level in parent 1 turned out a significant positive predictor of early attention effect. Removing additional predictors did not improve the model.

**TABLE 6:** Model comparison early selective attention.

Predictor	First significant early attention model			Preferred early attention model				Reduced early attention model		
	Adjusted *R*^2^ = 0.87			Adjusted *R*^2^ = 0.89				Adjusted *R*^2^ = 0.77		
	RSE = 2.19 (87 DF)			RSE = 2.19 (88 DF)				RSE = 2.21 (89 DF)		
	*p* < 0.05			*p* < 0.05				*p* < 0.05		
	β	SE	*p* <	β	SE	*p* <	95% CI	β	SE	*p* <
Intercept	−5.29	3.46		−4.31	3.26			−5.08	3.24	
Age	0.03	0.04		0.04	0.04			0.04	0.04	
**Swedish stronger language**	−2.95	1.16	0.05	−2.86	1.16	0.05	−5.16 to −0.56	2.53	1.14	0.05
**Education parent 1**	0.35	0.23		0.43	0.20	0.05	0.02–0.84	0.51	0.20	0.05
Education parent 2	0.16	0.18		0.65	0.43					
Income parent 1	0.72	0.44								
Income parent 2	−0.95	0.49		−0.84	0.47			−0.61	0.45	

*Standardized estimates, standard errors, and significance levels for predictors are included, as well as 95% confidence intervals for predictors in the preferred model. Adjusted *R*^2^ and residual standard error displayed for the full model and the preferred model. Significant predictors in bold script.*

For the late attention effect (mean difference in brain responses for attended versus unattended story in the time window 300–400 ms post stimulus onset), a full model was not significant and significance was first reached with a model including age, having Swedish as a stronger language, educational level, time/week at preschool, age at preschool enrollment and preschool quality, together explaining 11% of the variance in late attention effect. Reduction of the least contributing predictors led to additionally two models with very similar levels of explained variance and residual standard errors. Education in parent 2 positively predicted late attention. Further elimination of predictors made models slightly worse. See Table 7 for model comparison.

**TABLE 7:** Model comparison for late selective attention.

Predictor	First significant Late Attention Model			Preferred Late Attention Model				Reduced Late Attention Model		
	Adjusted *R*^2^ = 0.11			Adjusted *R*^2^ = 0.11				Adjusted *R*^2^ = 0.11		
	RSE = 2.0 (78 DF)			RSE = 1.99 (79 DF)				RSE = 2.0 (80 DF)		
	*p* > 0.05			*p* < 0.05				*p* < 0.05		
	β	SE	*p* <	β	SE	*p* <	95% CI	β	SE	*p* <
Intercept	−2.04	4.04		−1.30	3.95			−0.77	3.92	
Age	0.06	0.04		0.05	0.033			0.04	0.03	
Swedish stronger language	−1.66	1.25		−1.31	1.18					0.05
**Education parent 1**	−0.34	0.19		−0.30	0.19			−0.37	0.18	
**Education parent 2**	0.37	0.17	0.05	0.34	0.17	0.05	0.008–0.68	0.31	0.17	
Preschool time/week	−0.06	0.04		−0.06	0.04			−0.06	0.04	
Age at preschool start	0.06	0.04		0.06	0.04			0.06	0.04	
Preschool quality	−0.23	0.26								

*Standardized estimates, standard errors, and significance levels for predictors are included, as well as 95% confidence intervals for predictors in the preferred model. Adjusted *R*^2^ and residual standard error displayed for the full model and the preferred model. Significant predictors in bold script.*

## Discussion

In the current study we examined associations between aspects of language and EFs in a sample of 431 Swedish 4–6-year-olds as well as potential relations to age, sex, presence of other/additional languages than Swedish at home, parental SES and aspects of preschool attendance and quality. A subsample of 138 children participated in Swedish AudAt, an ERP experiment assessing selective auditory attention, hypothesized to be a neural correlate of EFs. In line with expectations, language and EF correlated significantly but we did not find any correlations between behaviorally assessed EF and selective auditory attention as assessed with ERPs. Age, sex and aspects of parental SES significantly predicted receptive vocabulary, morphosyntactic accuracy and EF. Selective attention was associated to parental education but not to age nor sex.

### RQ1. the Relationship Between Language Skills, EFs, and Auditory Selective Attention

Language and EF measures correlated significantly, but the correlations were to a large extent rather weak. Receptive vocabulary score showed the highest correlation with EFs, whereas measures of language extracted from children’s narratives correlated weakly to moderately with EF. Previous studies have often used vocabulary measures to represent “language” and the current results indicate that focusing solely on vocabulary may lead to overestimating the strength of the relationship between language and EF. BDS, considered an assessment of verbal working memory, was the EF measure that showed the strongest correlation to most language measures. It has been suggested that there is no functional separation between language processing and the capacity commonly referred to as verbal working memory (e.g., MacDonald and Christiansen, 2002). Such a statement may be seen as overly radical, but can nevertheless suggest that the associations in the current data between language measures and working memory may not be the most informative to shed further light on the language-EF relationship. The correlations among the different EF tasks were weak to moderate, indicating that the different tasks tap into different aspects of EFs.

Contrary to our hypothesis, the auditory selective attention measures did not show any significant relationships with any performance-based measure of EFs. Previous studies using the original AudAt paradigm have not used behavioral measures of EF (Coch et al., 2005; Sanders et al., 2006; Stevens et al., 2009; Neville et al., 2013; Hampton Wray et al., 2017). Nevertheless, it seems surprising that auditory selective attention was not associated with any EF test results in the current data, given the idea that selective attention may either be a foundation of EF or part and parcel of EF abilities, and empirical work indicating an association (e.g., Veer et al., 2017; Frick et al., 2018). In particular the lack of association between the attention affect and the Flanker task, which assesses interference control in the visual modality, is intriguing. However, it has been shown that auditory distractors are more difficult for young children to deal with than visual distractors (e.g., Robinson et al., 2018). Recent work has also shown modality differences for interference control in adults with dyslexia, suggested to reflect the importance of auditory selective attention for aspects of language such as speech processing and phonological awareness (Gabay et al., 2020, see also ten Braak et al., 2018). Further work is needed to investigate potential modality differences in selective attention and interference suppression during early childhood and the relation between AutAt and behaviorally assessed EF.

We found weak correlations between early attention effect and unified predicates and with morphosyntactic accuracy. Earlier work has suggested links between inhibition, which could include the ability to suppress irrelevant information, and aspects of morphosyntax (Ibbotson and Kearvell-White, 2015; Kaushanskaya et al., 2017; Gandolfi and Viterbori, 2020). Another possibility is that language somehow acts as a confounding factor in attention tasks, which has been shown to be the case in performance-based tasks (Victorino and Schwartz, 2015). It seems reasonable to believe that a listener facing the complex task to listen to two stories simultaneously, depends on both language skills and attentional skills to focus on one story and suppressing the other. Strong language skills may aid the child to attend one story over another, perhaps by making probabilistic predictions about linguistic events in the near future, and strong attentional skills may serve specifically to suppress unwanted information. Experiments with adults (e.g., Oberfeld and Klöckner-Nowotny, 2016) have indicated that variance in adult participants’ comprehension of speech in noisy environments could in part be explained by selective attention. In any case, potential links between children’s grammar skills, language comprehension and auditory selective attention need further consideration.

### RQ2. Associations Between Age, SES, Sex, and Bi/-Multilingualism and Language/EFs/Selective Attention

#### Age Associated With All Measures Except Selective Attention

Contrary to predictions, neither the early nor the late attention effect was significantly predicted by age. In contrast, all the behavioral and parent-rated measures were associated with age. An early study using the AudAt ERP paradigm also failed to find significant associations between attention effect and age (Coch et al., 2005). Receptive vocabulary score had the strongest correlation to age, suggesting that, although the PPVT is a somewhat problematic test due to the lack of Swedish official translation and norms, it reflects an expected increase in receptive vocabular as children grow older.

#### Socioeconomic Status Associated With Language, EFs, and Selective Attention

In accordance with our predictions, aspects of SES were significantly associated with receptive vocabulary, morphosyntax, EF composite and attention, although the current sample was skewed toward higher SES which may reduce the differential sensitivity to SES effect. For selective attention, levels of explained variance were low for both early and late attention effect. However, partly in line with previous studies and our hypothesis, aspects of SES (parental education) significantly predicted the early attention effect.

#### Associations to Bi-/multilingualism

The multilingual children did not perform on par with monolingual peers with regard to Swedish receptive vocabulary when controlling for SES. Bi- or multilingual children do not necessarily exhibit a gap in receptive vocabulary compared with monolingual children (Thordardottir, 2011), but our result is in line with outcomes in a large-scale Danish study on preschool-aged children, in which language skills of native Danish and immigrant children were compared (Højen et al., 2019). Immigrant children scored significantly lower than non-immigrant children on standardized language tests when controlling for SES, leading to the conclusion that measures should be taken to reduce inequalities in educational outcomes already in preschool, focusing on L2 language skills (ibid.). In our data, aspects of the child’s language situation with regard to stronger language and/or bilingualism did not explain EF variance, which could suggest that the EF tasks did not disfavor children who did not have Swedish as a first language. A curious finding was that having Swedish as a stronger language was a negative predictor of early attention effect. The challenging task of selectively listening to a narrative may require a child who is less proficient in the majority language to allocate more attentional resources to the task compared to a peer with stronger language skills, but further investigation, including gathering more data regarding the language situation for bi-/and multilingual children, is needed to see if this result replicates.

#### Possible Female Advantage

No specific predictions were made with regard to possible differences between girls and boys, given that previous results are diverging. Male sex was a negative predictor of receptive vocabulary score, morphosyntactic accuracy and EF composite score. Current results are thus in line with studies that suggest a female advantage for both language and EF. Language and EF differences between girls and boys are often explained by theories that stress the influence of social environment on language as well as other cognitive domains (see e.g., Eriksson et al., 2012, for a summary), for instance that parents expect different behaviors from girls and boys and interact differently depending on the child’s sex (e.g., Wanless et al., 2013). When it comes to gender equality, Sweden regularly ranks among the top countries (see e.g., United Nations Development Programme, n.d.). Nevertheless, child-rearing and pedagogical practices in relation to children’s gender and cognitive development could be further explored in the Swedish context.

#### Aspects of Preschool Attendance

Aspects of preschool attendance and quality were included in regression models primarily to control for variation. We found no significant effects of preschool quality but age at preschool start was a negative, albeit not significant predictor of EF score. Loeb et al. (2007) conducted a large study in the United States, attempting to find out what would be the ideal age for children to start daycare/preschool/nursery school. They found greater gains in prereading and math skills in children who started center care between ages 2 and 3, whereas starting earlier than age 2 was related to negative social effects (ibid.). Potential effects of age at preschool enrollment on children’s individual cognitive development needs further attention, not least since there may be complex interactions between age at preschool start, family SES and home situation.

#### Low Levels of Explained Variance for Morphosyntactic Accuracy and Selective Attention

For morphosyntactic accuracy, the proportion of explained variance was low, highlighting the need to investigate language in a broader sense than focusing solely on aspects of vocabulary, which has often been the case in previous studies showing associations between, for instance, language and SES (e.g., Hart and Risley, 1995; Geoffroy et al., 2007). Previous work has indeed indicated that individual differences in language ability to a large extent is due to genetic factors (see e.g., Stromswold, 2001, for a review) and has also revealed an increase in heritability of language skills from early to middle childhood (e.g., Hayiou-Thomas et al., 2012). For selective attention, levels of explained variance were also low. Earlier work using the original AudAt paradigm has primarily investigated selective attention in lower-SES samples (e.g., Stevens et al., 2009; Neville et al., 2013; Hampton Wray et al., 2017). In a rather homogeneous sample with regard to SES such as the current, genetics may play a bigger role than environmental factors in explaining variance in attention.

### Methodological Issues

Contextual factors can have a large impact on children’s performance in highly controlled experiments – for instance it has been shown that children’s performance on tasks assessing so called “hot” EFs, such as delay of gratification, is highly sensitive to factors such as group norms (e.g., Doebel and Munakata, 2018), and to which extent children find the test leader trustworthy (e.g., Ma et al., 2018). Such factors are difficult to entirely control and may have potential impact on the results’ general implications. The speakers who recorded the stories in Swedish AudAt were asked to read the stories with the same level of engagement and character speech as they would in a real-life situation, reading aloud to a preschooler. This may have driven bottom-up, stimulus-driven attentional processes to a larger extent than in the original AudAt. However, the original AudAt could hardly be interpreted as a pure measure of endogenous attention, as the probe sounds “ba” and “bz” are likely to attract stimulus-driven attention. Additionally, the images displayed on a screen during the experiment may drive bottom-up visual attentional processes. Several researchers have put forward the idea that endogenous and exogenous attention systems interact during real-time prioritization of attentional focus, especially in tasks requiring some kind of vigilance (e.g., Corbetta and Shulman, 2002; Maclean et al., 2009), and if an experiment should be considered ecologically valid, such interactions may be difficult to control/avoid completely. With respect to the subsample for AudAt, it should also be noted that there was an element of self-selection in the sampling procedure, since children themselves had the opportunity to decline participation. While such a procedure fulfills ethical requirements and gives agency to the child, it may have led to an ERP subsample that was not entirely representative of the full sample. Another methodological aspect is that the reliability of ERP components has relatively seldom been reported in previous work, which is remarkable considering how widely ERP measures are used in research (see also Huffmeijer et al., 2014).

Another potential methodological shortcoming concerns the parental questionnaire. Our desire to formulate the background questions in a way that would not discriminate non-traditional families had the downside that we cannot make any conclusions regarding the relative importance of maternal and paternal educational level and/or income.

### Future Work

There is a need for future empirical studies as well as theoretical work to further clarify the associations between language and EF, including the role of selective auditory attention, in children. Such an endeavor should attempt at recruiting children from diverse SES backgrounds and to follow participants longitudinally. It seems vital to administer an array of both language and EF tasks to reveal any specific links between language and EF skills, however there is a clear need for further development and validation of suitable assessment materials. In the Swedish context, in which a majority of children attend preschool more or less full-time from 1 to 2 years of age, effects on cognitive development of preschool attendance in general and of specific pedagogical practices need further investigation. Future work should preferably be based on and/or contribute toward a theoretical framework that is more informative than merely stating that strong skills within one cognitive domain is associated with, and/or leads to strong skills within another. Existing theories of EF are not always explicit with regard to potential connections to language. Exceptions include Barkley’s (1997) model of inhibition, sustained attention and EFs, which includes internal speech as an EF, and models put forward by Zelazo and colleagues (e.g., Cunningham and Zelazo, 2007; Zelazo, 2015), suggesting that EF is verbally mediated. Recent work by Gandolfi and Viterbori (2020) suggests that high levels of interference suppression may aid a child to develop their lexicon, both receptively and productively, but the authors also show that it is the ability to suppress irrelevant stimuli, rather than other forms of inhibition which is linked to, and may even predict, grammar skills. However, existing theories seem underspecified with regard to causal links between or common mechanisms in language and EF.

## Conclusion

In the current study, we confirmed links between language and EFs in Swedish children aged 4–6, although the strength of the relationship seems to be less pronounced if including measures of morphosyntax instead of focusing solely on vocabulary. Results confirmed a female advantage and associations to age and SES for both language and EF, whereas for auditory selective attention, only links to parental education were confirmed. Contrary to expectations we did not find associations between behaviorally assessed EF and selective auditory attention measured with ERPs. The current findings provide some evidence of links between selective attention and aspects of morphosyntax, and between working memory and language measures in general, but further work is needed to clarify the nature of the language–EF relationship.

## Data Availability Statement

The raw data supporting the conclusions of this article will be made available by the authors, without undue reservation.

## Ethics Statement

The studies involving human participants were reviewed and approved by the Regionala etikprövningsnämden [Regional ethics board], Karolinska Institute, Stockholm. Written informed consent to participate in this study was provided by the participants’ legal guardian/next of kin.

## Author Contributions

ST and PK contributed to the conception and design of the study. ST performed the statistical analysis. PK organized and pre-processed the ERP data. ST wrote the first draft of the manuscript. PK and FL wrote the sections of the manuscript. All authors contributed to manuscript revision, read and approved the submitted version.

## Conflict of Interest

The authors declare that the research was conducted in the absence of any commercial or financial relationships that could be construed as a potential conflict of interest.

## Footnotes

1 https://ki.se/en/orgid/303872, DNR nr: 2015/1664–31/5.

2 For activeTwo and CMS/DRL details, see http://www.biosemi.com/

3 https://tla.mpi.nl/tools/tla-tools/elan/

4 The parental questionnaire was gender-neutral in order not to discriminate against non-binary parents or families with same-sex parents.
